# Development of a conflict-free unsignalized intersection organization method for multiple connected and autonomous vehicles

**DOI:** 10.1371/journal.pone.0249170

**Published:** 2021-03-30

**Authors:** Qinglu Ma, Shu Zhang, Qi Zhou

**Affiliations:** Chongqing Jiaotong University, Nanan Distr., Chongqing, China; University of Shanghai for Science and Technology, CHINA

## Abstract

An effective traffic control strategy will improve travel reliability in urban transportation networks. Lack of coordination between vehicles, however, exacerbates congestion due mainly to frequent stops at unsignalized intersections. It is beneficial to develop a conflict-free cooperation method that collects basic safety message from multiple approaching Connected and Autonomous Vehicles (for short, CAVs) and guarantees efficient unsignalized intersection operations with safe and incident free vehicle maneuvers. This paper proposes an interspersed traffic organization method under controlled constraints. Firstly, relied on shared location technology and considered the operating characteristics of CAVs at unsignalized intersections to detect and analyze traffic conflicts to establish a right-of-way judgment model for CAVs. In order to further ensure the safety and operating efficiency of the vehicle, based on the judgment results of right-of-way judgment model, a vehicle speed guidance model is established for different traffic conditions. Taking the real city standard intersection as the experimental analysis object, through data collection and simulation experiment, the signal control method and the organization method proposed in this paper are compared and analyzed. The results showed that the traffic organization method proposed in this paper improves the operational efficiency of 46%, the average travel time is reduced by 6.54s, which is not only better than the signal control method, but also supports the development of car networking technology.

## 1. Introduction

With the development of society and economy, the extraordinary growth of the vehicle population has brought about increasing traffic control pressure [1]. The increasing trend in vehicle ownership is also an element caused traffic problems. Intersections as the bottleneck in the road network, have a lot of difficulties in traffic control remind to be solved. From the traffic accident data published in various regions, it can be seen that the proportion of accidents at intersections is large. For example, the data published in China showed that the number of accidents at intersections accounts for 30% of the total number of accidents, 36% of such data displays in the United States, 43% in Europe and 42.2% in Japan. Increasing signal lights is used for keeping the traffic sequence widely, which could relieve local traffic jam and at the same time lead to the vehicle delays in the whole road network increase gradually. In recent years, communication technologies and the internet of things provide a good solution for unsignalized intersections. The communication technologies can make it possible that allow all the vehicles enter the intersections to exchange the basic information. Many researchers have carried out intensive study on traffic organization of unsignalized intersections based on vehicular networking technology. Based on multi-agent self-driving cars, a control method [2] that can avoid vehicle collisions is designed using a phase-like method. The correctness and safety of the algorithm is proved through high-level petri net analysis. However, this method does not consider the vehicle’s motion sequence in terms of time, and has very strict requirements for the definition of intersection time and space, and the possibility of vehicle collision is relatively high. For the further more research, a first-come-first-serve algorithm [3] was proposed. And the intersection controller grants or rejects vehicle requests to proceed through the intersection once the requests are received. A heuristic optimization algorithm based on game theory [4] for unsignalized intersections. And proposed a self-adaptive cruise control system based on the algorithm. The experimental results showed that the system can reduce the total delay of the intersection. With the development of information technology, there are more and more possibilities for the information exchange between the CAVs. In order to further improve the efficiency of intelligent networked vehicles, some scholars will focus on the real-time collection of vehicle motion information. The centralized dynamic programming method [5] is used to find the movement track of each vehicle at the intersection at each discrete time. The computational complexity of the problem increases exponentially with the number of vehicles, so the approximate optimal solution cannot be found in real time. A centralized mixed-integer liner programming (MILP) intersection control model [6], where location and speed of autonomous vehicles in each time step are gathered to provide the shortest and longest travel times to reach an intersection. This method is also computationally complex and only addresses through movements. And on the basis of the reservation-based intersection controls, the improved methods are put forward. For guarantying driving safety [7] while improving the unsignalized intersection management efficiency. Dividing the intersection zone into different collision sections (CSs), formulating the intersection collision-free model into an absolute value programming (AVP) problem, which is proved to be NP-hard. Further, proposed an alternately iterative descent method (AIDM) to solve the AVP problem by assigning the optimal entering time for each arriving AV. In order to expand the vehicle capacity, a signal-head-free intersection control logic [8] is formulated into a dynamic programming model that aims to maximize the intersection throughput. A stochastic look-ahead technique is proposed based on Monte Carlo tree search algorithm to determine the near-optimal actions (i.e., acceleration rates) over time to prevent movement conflicts. The numerical results confirm that the proposed technique can solve the problem efficiently and addresses the consequences of existing traffic signals. And has gone a step further proposed distributed cooperative control logic [9] to determine conflict-free trajectories for CAVs in unsignalized intersections. The cooperative trajectory planning problem is formulated as vehicle-level mixed-integer non-linear programs (MINLPs) that aim to minimize travel time of each vehicle and their speed variations, while avoiding near-crash conditions. For minimizing the travel time and driving smoothness (longitudinally and laterally), and the terminal cost, which penalizes the deviations from the desired final state., a novel method [10] to model the trajectories of vehicles in two-dimensional space and speed was proposed. Which is based on optimal control theory, it assumes drivers schedule their driving behavior, including the steering and acceleration. Aiming to solve the problem of existing models’ complexity and information redundancy [11] proposed a queue length sensing model based on vehicle to everything (V2X) technology, which consists of two sub-models based on shockwave sensing and back propagation (BP) neural network sensing. Simulation results showed that the sensing accuracy of the combined model is proportional to the penetration rate of connected vehicles, and sensing of queue length can be achieved even in low penetration rate environments. About data fusion problem, a robust positioning approach [12] which considering track-to-track matching and fusion of position information obtained from multiple on-board sources such as GPS receiver, vehicle-to-vehicle (V2V) communication and automotive radar. Realistic 3D road traffic and wave propagation model is developed using a ray-tracing tool and the effectiveness of the proposed concept was evaluated. The system concept is validated by conducting extensive simulation considering realistic car following model. And the results showed that the method exhibits significant improvement in terms of positioning accuracy. A systematic approach [13] to the cooperation of connected vehicles at unsignalized intersections without global coordination. A task-area partition framework is proposed to decompose the mission of cooperative passing into three main tasks, i.e., vehicle state observation, arriving time optimization, and trajectory tracking control. And a distributed control algorithm is proposed to address parameter mismatches and acceleration saturation for fixed-time trajectory tracking control. To explicitly factor information flow topologies (IFT) dynamics and to leverage it to enhance the performance of cooperative adaptive cruise control (CACC) strategies [14], proposed the idea of dynamically optimizing the information flow topologies IFT for cooperative adaptive cruise control CACC, labeled the CACC-OIFT strategy. Under CACC-OIFT, the vehicles in the platoon cooperatively determine in real-time which vehicles will dynamically deactivate or activate the “send” functionality of their V2V communication devices to generate IFTs that optimize the platoon performance in terms of string stability under the ambient traffic conditions. The effectiveness of the proposed CACC-OIFT is validated through numerical experiments in NS-3 based on NGSIM field data, and the experiments proved that the proposed method is effective. For CAVs in multi-intersection road networks [15] proposed a cooperative autonomous traffic organization method. The methodological framework consists of threefold components: an autonomous crossing strategy based on a conflict resolution approach at unsignalized intersections, multi-objective trajectory optimization in road segments, and a composite strategy for route planning considering heterogeneous decision-making behaviors of CAVs based on social and individual benefit, respectively. extensive simulation experiments have been performed to demonstrate its advantage over conventional baseline schemes in terms of global traffic efficiency. A model-based optimization procedure for the design of a control system with signal synchronization was introduced [16], real-time bus priority and green light speed advisory to car drivers. The traffic model simulates car traffic as platoons and bus movements individually.

The above research results show the effectiveness of intelligent network technology in solving intersection conflict problems. These findings are of great significance to the CAV organization method of unsignalized intersections, but in general, there is a lack of comprehensive consideration of safety, traffic efficiency and CAV computing capacity. Therefore, this paper proposes the CAVs organization method for unsignalized intersections with the goal of improving the traffic efficiency at intersections and ensuring the driving safety of vehicles. The structure of this paper is arranged as follows: Section 2 introduces organization method of research condition and describes concretely how the vehicle can pass through the intersection safely and efficiently according to the real-time position, speed, track and other information of the vehicle. Section3 evaluates the performance of this method by comparing it to signalized intersection. Section 4 concludes the paper.

## 2. Traffic organization method of unsignalized intersection based on CAVs location service

### 2.1 Control area

**Fig 1:**
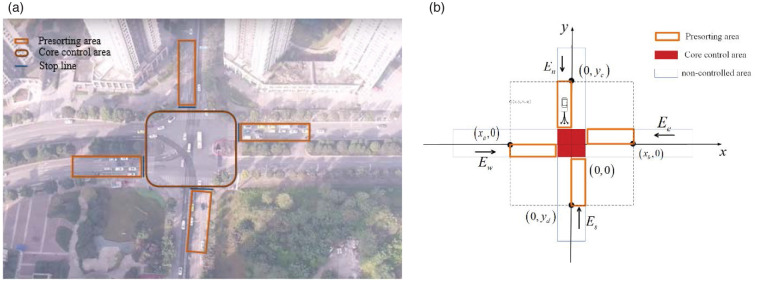
Control area divisions of an intersection. (a) Example of control area at unsignalized intersections (b) Control area divisions of an intersection. Photos by Shu Zhang, Processed by Shu Zhang. Fig 1(a) is similar but not identical to the original image and is therefore for illustrative purposes only.

The method proposed in this paper is based on the road being full of CAVs According to the shared location technology of CAVs, a typical intersection as shown in Fig 1(a) is established at the intersection and the coordinate system is established based on the center point of the intersection of the north-south road and the east-west road as the coordinate origin. As shown in Fig 1(b), take the east-west road as the x-axis, and take the north-south road as the y-axis. and *E*_*w*_, *E*_*e*_, *E*_*n*_ and *E*_*s*_ represent the four entrances of west, east, south and north, and sets a certain range at the intersection as the sensing area Ω, that is Ω = Ω = {(*X*, *Y*).*X* ∈ (*X*_*a*_, *Y*_*b*_), *Y* ∈ (*T*_*c*_, *Y*_*d*_)} In order to realize the distributed traffic organization method for CAVs at unsignalized intersection, the research scope is further divided into presort area, core control area and non-controlled area.

**Fig 2:**
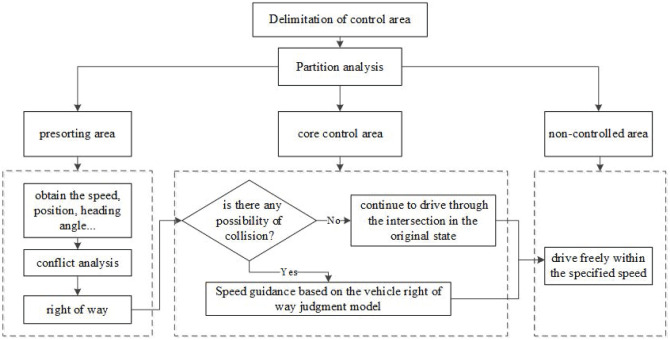
Control area divisions of an intersection.

When the vehicle (*C*_*i*_) enters the presorting area, the background control center will continuously obtain the speed(v_*i*_), position (*x*_*i*_, y_*i*_), heading angle(*φ*_*i*_) and other information of the vehicle at any time, and predict whether there is a conflict between vehicles according to this information. As shown in Fig 2, if there is no possibility of collision between vehicles, the vehicle will continue to drive through the intersection in the original state; if there is possible conflict, it will be based on the vehicle right of way judgment model in advance, and then the *C*_*i*_ is guided to pass through the intersection conflict area safely and quickly according to the speed guidance strategy. The *C*_*i*_ will always drive in the fixed lane in the presorting area, and there will be no abnormal driving behaviors such as lane changing and overtaking. All vehicles enter the core control area of the intersection when passing through the stopping line. Core control area is the most frequent area where conflicts occur at intersections. In order to ensure driving safety, proposed a method to guide the vehicle generally runs at a constant speed or decelerates in this area. After passing through the core control area, the vehicle enters the free driving area, and the vehicle can drive freely within the specified speed until it leaves the intersection control area.

According to the shared location theory of CAVs, considering the traffic flow and average vehicle speed, set the control area near the intersection, and study the size, feasibility, and applicability of the set area. By formulating the basic flow of the control strategy for unsignalized intersections, the control area is divided into three parts: the presorting area, the core control area, and the non-controlled area. The presorting area and the core control area is the focus of this article.

### 2.2. Organization and coordination method for unsignalized intersections

#### 2.2.1. Determination of vehicle priority

**Fig 3:**
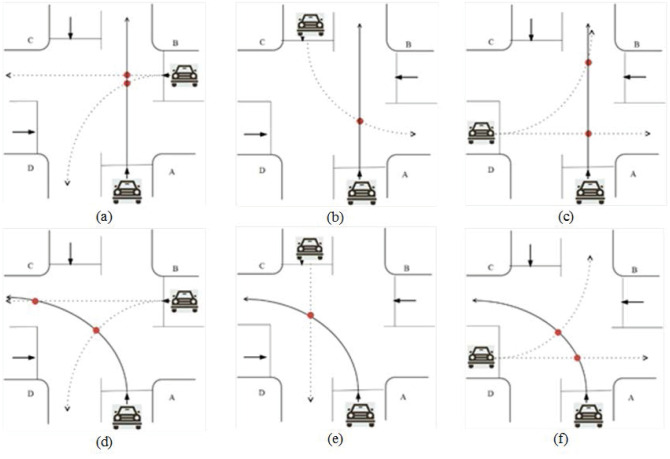
(a) Go straight with entrance B; (b) Go straight with entrance C; (c) Go straight with entrance D; (d) Turn left with entrance B; (e) Turn left with entrance C; (f) Turn left with entrance D.

Vehicle conflict coordination at unsignalized intersections is mainly through controlling the right of way of vehicles on different approaches to reduce the vehicle conflict points from the vehicle traffic sequence. After obtaining the priority, vehicles can pass through the intersection safely without collision. Before the right of way is allocated, it is assumed that there is no conflict of any kind between vehicles with the right of way at the same time. For a simple intersection as showed in Fig 3, the following points of collision that may occur when vehicles turn left and go straight.

When the CAV enters the control area of the unsignalized intersection, the real-time position, speed, trajectory and other driving information among the vehicles are shared. The signal intersection traffic conflict coordination mainly through the control of different entrance lane traffic, therefore, after entering the control area per vehicle will be based on their movement speed and trajectory information to calculate the time of arrival in the conflict, and sharing the results to other vehicles, based on the shared data between the vehicle will appear on the intersection of the sort results.

**Fig 4:**
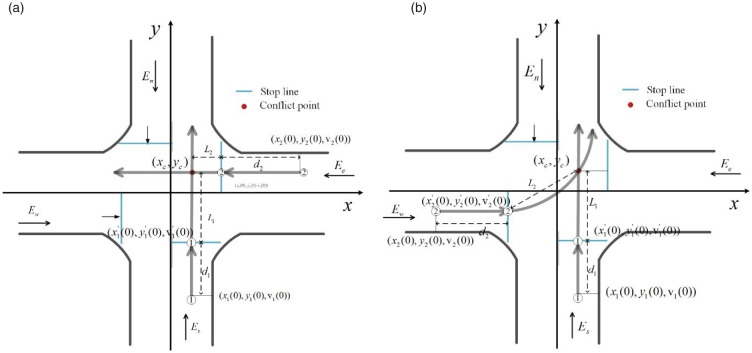
(a) Distance Analysis of straight conflict point; (b) Distance Analysis of left-turn conflict point.

As shown in Fig 4, after entering the control area of the intersection, the conflicting southbound and eastbound straight vehicles will share their position, speed and other motion information between the vehicles according to the shared location technology of the Internet of Vehicles. The vehicle background core compares its travel time based on the obtained movement information of other vehicles, and finally obtains the judgment result of the road right of way model. For the reader’s convenience, a list of the most important variables used in the algorithm is presented in Table 1.

**Table 1:** List of most important variables.

*v*_*i*_(0)	The instantaneous velocity of the connected/autonomous vehicles entering the sensing region
(*x*_*i*_(0), *y*_*i*_(0))	The position coordinate of vehicle *c*_*i*_ entering the control area
$\left(x_{i}^{\prime }(0),y_{i}^{\prime }(0)\right)$	The position coordinate of vehicle *c*_*i*_ arrived at the stop line
(*x*_*c*_, *y*_*c*_)	The position of conflict points
*d*_*i*_	The distance from the current car to the stopping line
*t*_*a*,*i*_	The time of vehicle arrives at the intersection conflict zone
*t*_*b*.*i*_	The time of vehicle arrives at the stopping line
*t*_*i*_	The total movement time of the vehicle from the position of entering the control area to the collision point
*L*_*i*_	The distance of vehicle from the stopping the conflict point to the conflict point
*a*_*i*_	Constant acceleration

Note: *i* = 1,2…n where n represents the number of vehicles entering the intersection control area.

According to the kinematics equation $v_{i}(0)t_{a}+\frac{1}{2}a_{i}t_{a}{}^{2}=d_{i}$, we can get the time *t*_*a*,*i*_ when the vehicle reaches the stopping line, the time *t*_*b*,*i*_ from the stopping line to the conflict point and the distance *L*_*i*_ between stopping line and the conflict point can be calculated as:

$$\left\{ \begin{array}{l} t_{a,i}=\frac{-v_{i}(0)+\sqrt{{\left[v_{i}(0)\right]}^{2}+2a_{i}d_{i}}}{a_{i}}\\ t_{b,i}=\frac{L_{i}}{v_{i}(0)+a_{i}t_{a}{}_{,i}}\\ L_{i}=\sqrt{{\left(x_{c}-x_{i}^{\prime }(0)\right)}^{2}+{\left(y_{c}-y_{i}^{\prime }(0)\right)}^{2}} \end{array}\right.$$ (1)

The total movement time of the vehicle from the position of entering the control area to the collision point can be calculated as:

$$t_{i}=t_{a,i}+t_{b,i}=\frac{-v_{i}(0)+\sqrt{{\left[v_{i}(0)\right]}^{2}+2a_{i}d_{i}}}{a_{i}}+\frac{\sqrt{{\left(x_{c}-x_{i}^{\prime }(0)\right)}^{2}+{\left(y_{c}-y_{i}^{\prime }(0)\right)}^{2}}}{v_{i}(0)+a_{i}t_{a}{}_{,i}}$$ (2)

In order to determine the order of arrival to the conflict area in advance, we arranged the time for vehicles to arrive at the conflict area at the intersection from small to large, and constructed a virtual platoon to project these vehicles onto the virtual lane. Then the right of way is determined by taking into account the actual situation of all vehicles arriving at the stop line, as well as information such as current position, speed, heading angle and destination position. The control center will judge whether there will be a collision between vehicles based on the current running trajectory of vehicles, and presorting vehicles based on the collected speed, acceleration, position, target lane and other driving state information to ensure the safety of vehicles at the intersection.

#### 2.2.2. Straight-through conflict guidance speed analysis

**Fig 5:**
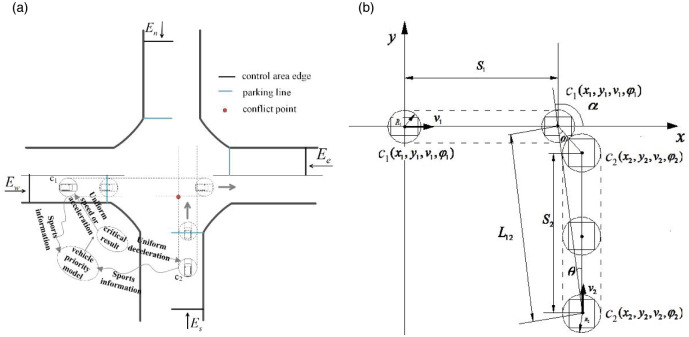
(a) Simplified schematic diagram of straight-through conflict; (b) Velocity analysis.

After the vehicle has determined the right of way, in order to further ensure the safety of the autonomous vehicle in the core control area, two possible conflicts will be selected, going straight and turning left, based on the speed, position and heading angle of the critical state of the collision between the two vehicles. Information is used to determine the speed range of the vehicle in the core control area, so as to guide the speed of the autonomous vehicle in the core control area. Taking the *c*_1_ (*E*_*w*_ − *E*_*e*_) and the *c*_2_ (*E*_*s*_ − *E*_*n*_) as an example, as shown in Fig 5(a), assuming that the vehicle *C*_1_ obtains the priority, the vehicle *C*_1_ can choose to pass through the intersection at a constant speed or accelerate, and the vehicle *C*_2_ must decelerate to pass.

When vehicle *C*_2_ obtaining the right of way, *C*_2_ can choose to accelerate or pass through the intersection at a constant speed, so as to model *C*_1_ and *C*_2_ analyze the speed as shown in the Fig 5(b).

When the centre of mass of vehicle *C*_1_ and vehicle *C*_2_ is connected, the straight line is

$$\left\{ \begin{array}{l} L_{12}=\sqrt{{\left(y_{2}(t)-y_{1}(t)\right)}^{2}+{\left(x_{2}(t)-x_{1}(t)\right)}^{2}}\\ v_{1}{}_{x}(t)=v_{1}(t)\text{cos}\varphi_{1},v_{1}{}_{y}(t)=v_{1}(t)\text{sin}\varphi_{1}\\ v_{2}{}_{x}(t)=v_{2}(t)\text{cos}\varphi_{2},v_{2}{}_{y}(t)=v_{2}(t)\text{sin}\varphi_{2} \end{array}\right.$$ (3)

The relative velocity difference (Δ*v*) and the time to conflict (*TTC*) of *C*_1_ and *C*_2_ collisions is obtained:

$$\Delta v=v_{2}(t)-v_{1}(t)=\sqrt{{\left(v_{2}(t)\text{cos}\varphi_{2}-v_{1}(t)\right)}^{2}+{\left(v_{2}(t)\text{sin}\varphi_{2}\right)}^{2}}$$ (4)

$$TTC=\frac{L_{12}}{\Delta v}=\frac{\sqrt{{\left(y_{2}(t)-y_{1}(t)\right)}^{2}+{\left(x_{2}(t)-x_{1}(t)\right)}^{2}}}{\sqrt{{\left(v_{2}(t)\text{cos}\varphi_{2}-v_{1}(t)\right)}^{2}+{\left(v_{2}(t)\text{sin}\varphi_{2}\right)}^{2}}}$$ (5)

When the vehicle *C*_1_ passes through the conflict area at a constant speed, the moving distance of vehicle *C*_1_ is *S*_1_:

$$\left\{ \begin{array}{l} t_{1}=S_{1}/v_{1}(t)\\ 0\leq t_{1}\leq TTC \end{array}\right.$$ (6)

The range of speed can be obtained as follows:

$$\frac{L_{12}}{\sqrt{{\left(v_{2}\text{cos}\varphi_{2}-v_{1}\right)}^{2}+{\left(v_{2}\text{sin}\varphi_{2}\right)}^{2}}}\leq v_{1}\leq V_{restrict}$$ (7)

When *C*_1_ uniformly accelerates through the conflict area with acceleration *a*_1_, the time required is *t*_1_:

$$\left\{ \begin{array}{l} t_{1}=\frac{-v_{1}(0)+\sqrt{{\left[v_{1}(0)\right]}^{2}+2a_{1}S_{1}}}{a_{1}}\\ 0\leq t_{1}\leq TTC \end{array}\right.$$ (8)

The range of velocity can be obtained as follows:

$$v_{1}(t)\geq \sqrt{{\left[v_{1}(0)\right]}^{2}+2a_{1}S_{1}}$$ (9)

It can be concluded that the speed range of vehicle *C*_1_ is:

$$\{\sqrt{{\left[v_{1}(0)\right]}^{2}+2a_{1}S_{1}}\}\cap \{\frac{L_{12}}{\sqrt{{\left(v_{2}\text{cos}\varphi_{2}-v_{1}\right)}^{2}+{\left(v_{2}\text{sin}\varphi_{2}\right)}^{2}}}\}\leq v_{1}(t)\leq V_{restrict}$$ (10)

The vehicle *C*_2_ must decelerate before the vehicle *C*_1_ passes through the conflict zone, and the determination of its guiding speed range must take the driving distance of the vehicle *C*_2_ into consideration. The restrictions are as follows:

$$\left\{ \begin{array}{l} v_{1}(t)\geq \sqrt{{\left[v_{1}(0)\right]}^{2}+2a_{1}S_{1}}\\ R_{1}=\frac{\sqrt{L_{1}{}^{2}+W_{1}{}^{2}}}{2}\\ R_{2}=\frac{\sqrt{L_{2}{}^{2}+W_{2}{}^{2}}}{2}\\ \frac{R_{1}+R_{2}}{\text{sin}\theta }=\frac{S_{2}}{\text{sin}\omega }=\frac{L_{12}}{\text{sin}(\pi -\theta -\omega )}\\ \frac{L_{12}\text{sin}(\alpha -\varphi_{2})}{R_{1}+R_{2}}=\text{sin}\theta \sqrt{1-{\left(\text{sin}\omega \right)}^{2}}+\text{cos}\theta \text{sin}\omega \end{array}\right.$$ (11)

Find the critical speed *v*_2_(*t*)_max_ at which vehicle *C*_2_ does not collide with vehicle *C*_1_:

$$v_{2}{\left(t\right)}_{\text{max}}=\sqrt{2a_{2}S_{2}+v_{2}{\left(0\right)}^{2}}=\sqrt{\frac{2a_{2}(R_{1}+R_{2})\text{sin}\omega }{\text{sin}(\alpha -\varphi_{2})}+v_{2}{\left(0\right)}^{2}}$$ (12)

The speed range that vehicle *C*_2_ needs to control to avoid collision with vehicle *C*_1_ is:

$$0\leq v_{2}(t)\leq \sqrt{\frac{2a_{2}(R_{1}+R_{2})\text{sin}\omega }{\text{sin}(\alpha -\varphi_{2})}+v_{2}{\left(0\right)}^{2}}$$ (13)

In summary, if vehicle *C*_1_ has priority over vehicle *C*_2_ to obtain the right of way, vehicle *C*_1_ and vehicle *C*_2_ can travel at uniform acceleration with accelerations *a*_1_, *a*_2_ (*a*_1_ and *a*_2_ can be 0) respectively. When vehicle *C*_1_ passes through the conflict zone and vehicle *C*_2_ happens to arrive, the two vehicles are in a critical state of collision. In order to ensure the safe passage of signal vehicle and the safe passage of the two vehicles, the speed of the two vehicles needs to be guided before this.

#### 2.2.3. Turning-left conflict guidance speed analysis

**Fig 6:**
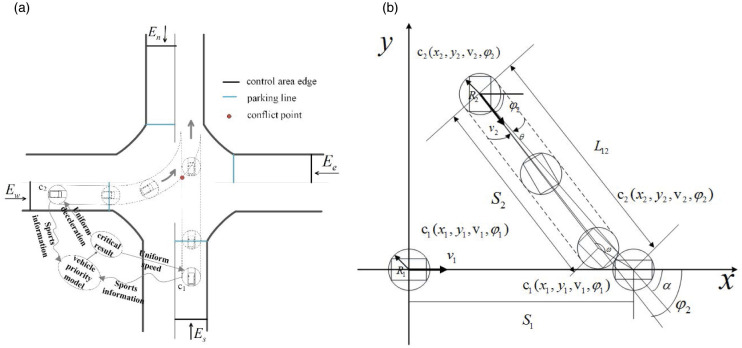
(a) Simplified schematic diagram of turning-left conflict; (b) Velocity analysis.

As shown in Fig 6(a), assuming that the vehicles have been judged the right of way before entering the intersection conflict area, if the vehicle *C*_1_ first passes the stopping line to reach the conflict area, the vehicle *C*_2_ needs to turn left through the intersection, according to the intersection traffic rules, when the vehicle *C*_2_ passes through the intersection, it generally needs to slow down and the vehicle moves approximately in a circular motion. Therefore, the measures taken for the two vehicles are that the vehicle *C*_1_ passes through the intersection at a constant speed, and the vehicle *C*_2_ decelerates or stops in front of the stopping line.

From formula (3)–(7), it can be calculated that the guidance speed range of the vehicle *C*_1_ in the straight direction that obtains the priority right of way in the left-turn conflict is:

$$\frac{\sqrt{{\left(y_{2}(t)-y_{1}(t)\right)}^{2}+{\left(x_{2}(t)-x_{1}(t)\right)}^{2}}}{\sqrt{{\left(v_{2}\text{cos}\varphi_{2}-v_{1}\right)}^{2}+{\left(v_{2}\text{sin}\varphi_{2}\right)}^{2}}}\leq v_{1}(t)\leq V_{restrict}$$ (14)

Vehicle *C*_2_enters the intersection conflict area after vehicle *C*_1_, and is decelerating until it reaches the conflict area with vehicle *C*_1_. After passing the stop line, it keeps making approximate circular motions. The maximum resultant force on the road surface is set as *F*_max_ and can be decomposed into longitudinal force and lateral force *F*_x_, according to the physics of circular motion:

$$\{\begin{array}{c} F_{\text{max}}=\mu_{\text{max}}mg=\sqrt{F_{x}{}^{2}+F_{y}{}^{2}}\\ F_{x}=m\frac{dv}{dt}\\ F_{y}=\frac{mv^{2}}{R_{0}} \end{array}$$ (15)

Where: *R*_0_ represents the turning radius of the road (obtained by the Internet of Vehicles);*m* represents car quality:*v* represents turning speed; *μ*_max_ represents the maximum static friction coefficient, generally, *μ*_max=0.9_, *g* = 9.8*m*/*s*^2^.

The longitudinal force *F*_x_ ignores the influence of various resistances during the car’s traveling process, and the lateral force *F*_x_ also ignores the influence of the lateral gradient of the road. On this basis, considering the vehicle parameters and road characteristics but not considering the possibility of the vehicle rolling over, when the longitudinal force *F*_x_ is equal to the maximum ground adhesion, the vehicle reaches the critical speed, that is, the maximum safe speed *v*_*critical*_ when the vehicle turns.

$$v_{critical}=k_{1}k_{2}\sqrt{\mu_{\text{max}}gR_{0}}$$ (16)

Where: *k*_1_ depends on the vehicle quality, the height of the center of mass of the vehicle, road conditions and other related parameters, *k*_2_ the safety optimization coefficient to improve driving safety, generally *k*_1_ = 0.6, *k*_1_ = 0.3.

Considering the travel distance of vehicle *C*_1_, the speed limit conditions are as follows:

$$\left\{ \begin{array}{l} R_{1}=\frac{\sqrt{L_{1}{}^{2}+W_{1}{}^{2}}}{2}\\ R_{2}=\frac{\sqrt{L_{2}{}^{2}+W_{2}{}^{2}}}{2}\\ R_{2,o}=R_{r}+\frac{D_{r}}{2}\\ \frac{R_{1}+R_{2}}{\text{sin}\theta }=\frac{L_{12}}{\text{sin}\omega }=\frac{S_{2}}{\text{sin}(\pi -\theta -\omega )} \end{array}\right.$$ (17)

The maximum distance that can be obtained for vehicle *C*_2_ is *S*_2_ and the critical speed *v*_2_(*t*) that does not collide with vehicle *C*_1_:

$$S_{2}=\frac{\left(R_{1}+R_{2})\text{sin}(\pi -\theta -\omega \right)}{\text{sin}\theta }$$ (18)

$$v_{2}(t)=\sqrt{2a_{2}S_{2}+v_{2}{\left(0\right)}^{2}}=\sqrt{\frac{2a_{2}(R_{1}+R_{2})\text{sin}(\pi -\theta -\omega )}{\text{sin}\theta }+v_{2}{\left(0\right)}^{2}}$$ (19)

It can be concluded that the speed range of vehicle *C*_2_ is:

$$0\leq v_{2}(t)\leq \{k_{1}k_{2}\sqrt{\mu_{\text{max}}gR_{0}}\}\cap \{\sqrt{\frac{2a_{2}(R_{1}+R_{2})\text{sin}(\pi -\theta -\omega )}{\text{sin}\theta }+v_{2}{\left(0\right)}^{2}}\}$$ (20)

## 3. Result of experimental simulation and analysis

**Fig 7:**
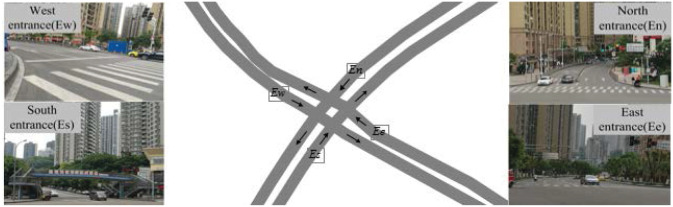
Present situation of intersection of Fenglin road and Rongqiao road. (The figure is similar but not identical to the original image and is therefore for illustrative purposes only).

This paper selects a planar crossroad of the Fenglin Road and Rongqiao Road of Nan’an District, Chongqing as an example to compare the traffic capacity of the unsignalized intersection and the signalized intersection under the same traffic conditions. In Fig 7, the Fenglin Road (*En* − *Es*) is a one-way two-lane road, and the two lanes are left, straight and right. Rongqiao Road (*Ew* − *Ee*) is a one-way three lane, and the three lanes contain left, straight, straight and right. Thus, the vehicle runs from the South (*Es*) to the West (*Ew*), that is the left turn collision is shown in Fig 2. The curve with arrowheads represents the track of the vehicle, and the intersection of the two curves represents the conflict point of the track of the vehicle. Taking Fenglin road and Rongqiao road as example, when the vehicle reaches the control area, it will automatically receive the movement tracks of other vehicles in the area, at the same time, the control centres of the vehicle will mark the conflict point of the vehicle according to the conflict model and feedback the mark result to the motion control area of the vehicle. For example, When the vehicle enters from the east entrance and is about to make a left turn, as shown in the conflict model, there will be four conflict points, at this time, the control centres of the vehicle will judge whether there are vehicles coming from the north entrance to turn left, the south entrance to go straight, and the left-turn, the west entrance to go straight according to the movement tracks of other vehicles received, combined with the vehicle traffic weight model, the calculation results are fed back to the vehicle’s motion control area.

Investigated the traffic flow and basic conditions at the intersection of Fenglin Road and Rongqiao road from 5:00 to 6:00 pm. According to the survey, the entrance of *Ee*, *Ew*, *En* all have crosswalk lines, while *Ew* has the pedestrian bridge. There are traffic lights at *En* − *Es* and *Ew* − *Ee* junctions, and more basic traffic information of Fenglin road and Rongqiao road is as Table 2.

**Table 2:** The of intersection of Fenglin road and Rongqiao road.

Direction of approach	*En*			*Ee*			*Es*			*Ew*		
Road condition	Two lanes			Three lanes			Two lanes			Three lanes		
Width of approach(*m*)	9			9			9			9		
Driving direction	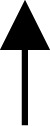	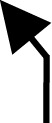	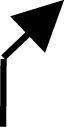	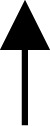	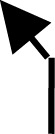	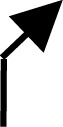	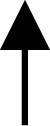	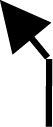	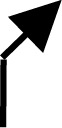	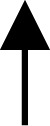	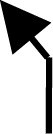	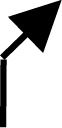
Flow(puc/*h*)	318	84	144	276	74	144	324	108	124	284	84	132

**Fig 8:**
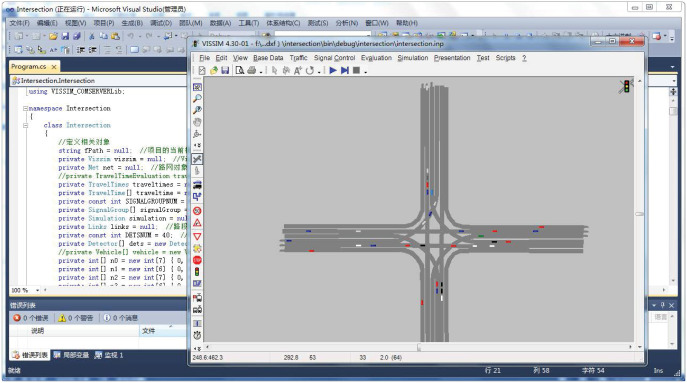
Visual studio calls VISSIM to compile the simulation result graph.

In order to verify the effectiveness of the strategy proposed in this article, the main purpose is to use Visual Studio simulation software for secondary development of VISSIM. The COM interface technology is used to realize the data exchange between the VISSIM simulation model and the logic control program of Visual Studio. Compile the unsignalized intersection control strategy studied in this paper through the Visual Studio control platform, as shown in Fig 8.

**Fig 9:**
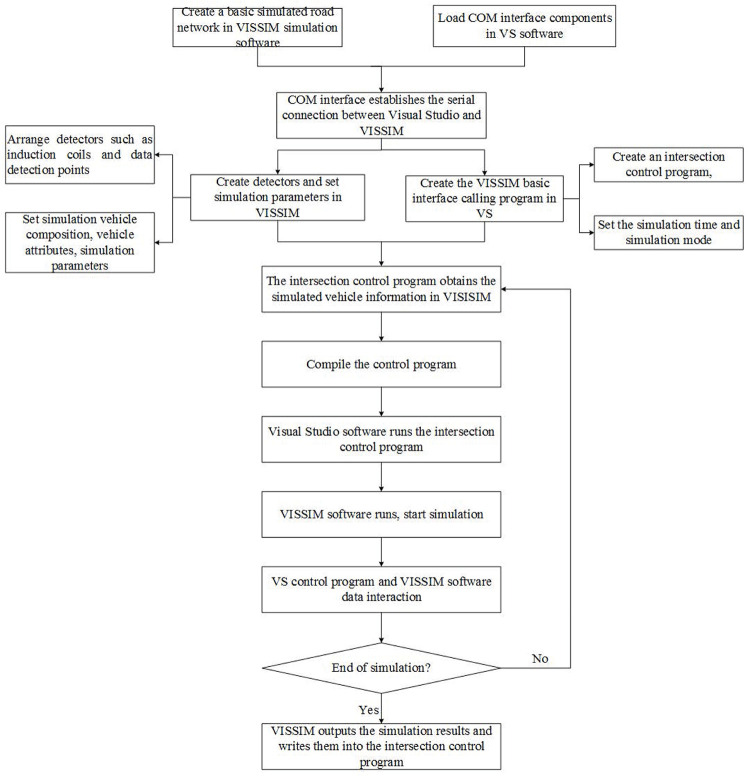
Simulation flow chart of control strategy for unsignalized intersection.

The verification platform based on Visual Studio programming software and VISSIM simulation software. In order to prove that the control strategy in this paper is safer and more effective than the actual signal control strategy, it is necessary to construct the same simulation environment as the actual signalized intersection. The control strategy is a control program written in C# language that can judge vehicle conflicts, traffic priority, and conduct vehicle speed guidance. The simulation process of the unsignalized intersection control strategy is roughly shown in Fig 9.

**Fig 10:**
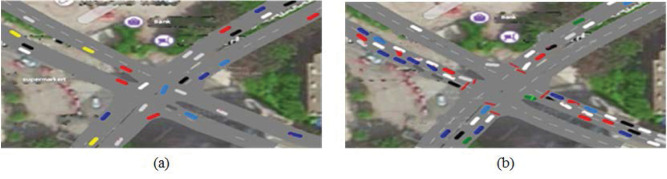
(a) Simulation of unsignalized intersection; (b) Simulate reality signalized intersection.

The effectiveness and feasibility of the proposed method are verified by evaluating the results of two simulation experiments with quantitative data indexes. The simulation is shown in Fig 10.

There are many aspects to evaluate the effect of traffic control, the main parameters are: traffic flow, traffic density, queue length, delay time, parking frequency, average speed and so on. In this paper, the average travel time and delay time are chosen as a representative evaluation standard, and the average travel time and delay time of each entrance road is calculated by two methods respectively, the simulation results are shown in Table 3.

**Table 3:** Comparison of simulation results.

flow direction	*Ew* − *En*	*Ew* − *Ee*	*Ee* − *Es*	*Ee* − *Ew*	*En* − *Ee*	*En* − *Es*	*Es* − *Ew*	*Es* − *En*
average travel time signalized intersection(s)	22.7	24.8	14.6	20.4	18.8	20.1	20.9	23.1
average travel time proposed method at un-signalized intersection(s)	13.1	14.0	11.7	18.5	13.0	13.5	15.9	14.5

The simulation results showed that under the same traffic conditions, this non-signal control method of traffic organization of intersection proposed by this paper reduced average travel time by 6.54 seconds and delay time by 3.375 seconds, traffic efficiency increased by 46% than the existing traffic organization. In order to verify the validity and correctness of the proposed traffic control method, Vissim 4.3 is used to simulate the actual traffic conditions at signalized and unsignalized intersections. In order to reduce the influence of different factors such as simulation parameters on the simulation results, the two control methods will simulate the traffic volume from 400 veh/h to 1000 veh/h in increments of 50 veh/h under the same simulation parameters as shown in Table 4.

**Table 4:** Simulation parameter table.

Simulation parameters	parameter values
*v*_max_/(*km*•*h*^-1^)	40
*a*_max_/(*m*•*h*^-2^)	2
*flow*/(*veh*•*h*^-1^)	400~1000
Simulation step size /s	1
Ratio of turning left/%	19
Ratio of going straight/%	55
Ratio of turning right /%	26
Time/s	3600
Other simulation parameters	Default value of Vissim

**Fig 11:**
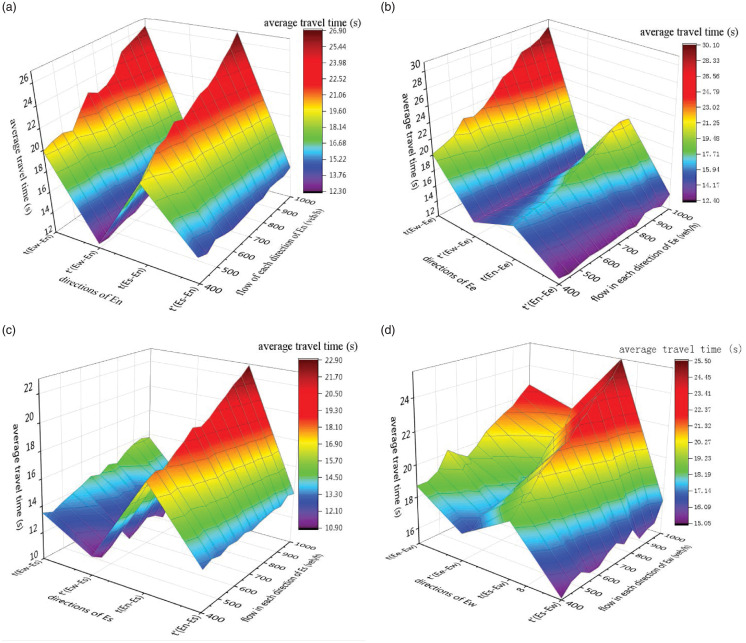
(a) Comparison of travel time at *En*; (b) Comparison of travel time at *Ee*; (c) Comparison of travel time at *Es*; (d) Comparison of travel time at *Ew*.

Simulate the different flow rates of the four entrances (*En*, *Ee*, *Es* and *Ew*)in the straight and left directions respectively. The average travel time was used as the simulation output index, and the simulation output results were plotted as a graph as shown in Fig 11, where *t* represents the signal control method and *t*′ represents the method proposed in this paper.

From Fig 11(a)–11(d), it can be seen that when the flow of the entrance road changes from 400 veh/h to 1000 veh/h, the average travel time of the four entrance roads without signal intersection is relatively stable. When the traffic volume of the entrance road for vehicles going straight at a signalized intersection is from 400 veh/h to 1000 veh/h, the average travel time is approximately linearly increased. For vehicles turning left at an intersection, the average travel time of vehicles at unsignalized intersections is relatively stable, while the average travel time of vehicles at signalized intersections fluctuates greatly with the traffic flow of the entrance lane, which is approximately for linear growth. It can be inferred from this that under the same conditions, the control strategy for unsignalized intersections is more stable than the control strategy for signalized intersections.

## 4. Conclusions

With the development of information technology and various kinds of sensors, the process of road intersection information control has been accelerated. The application of man-vehicle-road integrated vehicle network system in unsignalized intersection can not only avoid vehicle conflict, but also improve vehicle traffic efficiency and reduce environmental pollution. In this paper, the control method of CAVs under the vehicle network environment is studied, and the vehicle behavior control method is optimized on the basis of the existing control method of unsignalized intersection, the effectiveness of the proposed algorithm is verified by simulation. In the course of the research, this paper makes more assumptions, only studies the conflict relationship between different lanes at intersections and establishes a speed guidance model based on merging conflict and crossing conflict to solve the vehicle conflict problem, there is a certain deviation between the traffic condition and the running state of vehicles in the unsignalized intersection, so it needs further research.
